# Calendar Horizon as a Boundary Affordance: An Attempt-Centric Eye-Tracking Analysis of Calendar Scheduling Interfaces

**DOI:** 10.3390/jemr19020027

**Published:** 2026-03-02

**Authors:** Nina Xie, Yuanyuan Wang, Yujun Liu

**Affiliations:** 1 Department of Management, Faculty of Business, Lingnan University, 8 Castle Peak Road, Tuen Mun, Hong Kong; 2 Management and Strategy, Lee Shau Kee School of Business and Administration, Hong Kong Metropolitan, Homantin, Kowloon, Hong Kong; cyywang@hkmu.edu.hk; 3 Faculty of Business, Lingnan University, 8 Castle Peak Road, Tuen Mun, Hong Kong; yujunliu@ln.hk

**Keywords:** calendar interface design, eye tracking, visual process analytics

## Abstract

Digital calendars are interactive representations of time that shape both scheduling outcomes and the micro-process of searching, verifying, and revising candidate placements. We examine calendar horizon—whether weekend time is visible in the default week view—as a boundary affordance in scheduling interfaces. Using eye tracking and interaction logs, we model each scheduling episode as a sequence of placement attempts and align gaze to each attempt, partitioning it into Early/Mid/Late phases and summarizing attention across structural AOIs (task panel, calendar grid, and the weekend column when present). Two experiments used drag-and-drop and dropdown slot-picking; weekend visibility was manipulated within the dropdown interface, while evening slots remained available. Across 105 participants (1018 task episodes), AttemptsCount ranged from 1 to 7. AttemptsCount predicted gaze-based process cost: each additional attempt corresponded to ~56% more total fixation duration. Personal tasks required more attempts than work tasks and elicited stronger Late-phase weekend verification when the weekend was visible. Horizon cues also shifted boundary outcomes: hiding the weekend reduced weekend placements and increased reliance on evening scheduling, indicating displacement into adjacent time regions. These findings position calendar horizon as a design lever that shapes both process (verification) and outcomes (boundary placements), with implications for calendar UIs and mixed-initiative scheduling tools.

## 1. Introduction

Digital calendars are a primary interface through which people coordinate work, family, and personal life. CSCW research shows that calendar work is iterative: “making time” involves negotiation, breakdowns, and repair within social and organizational routines [1,2,3]. Recent work similarly frames calendar work as situated, intersecting with distributed coordination and shifting work–life practices [4,5].

This paper investigates a consequential design lever in calendar interfaces: the calendar horizon. By horizon, we mean the temporal scope that is made visible and treated as the default candidate space for scheduling—operationalized here as whether weekend time is shown alongside weekdays. We use “weekend” as the conceptually general boundary; in our experiments, the visible weekend region corresponds to a weekend column (Saturday in some conditions, and weekend days in others). Representational choices in calendar horizons are not neutral: research on time visualization and chrono-design argues that visual structures and partitions shape interpretation and normative judgments about time use [6,7,8].

From a boundary-management perspective, the default calendar horizon is an interface-level boundary setting: it shapes segmentation versus integration by making some time regions more cognitively salient and more socially “legitimate” as scheduling options [9,10,11]. Boundary-management scholarship emphasizes that work–non-work boundaries are enacted through everyday practices and artifacts, and that boundary permeability and flexibility vary across people and contexts [9,10,11,12]. We therefore treat horizon as a form of digital choice architecture: by defining what is visible on the default scheduling surface, it influences both (a) availability-based choice construction—salient options are more likely to be retrieved and compared during decision construction [13,14]—and (b) normative pressure—defaults communicate what is typical or “appropriate,” thereby steering behavior even when alternatives remain possible [15,16,17]. Applied to weekend visibility, this implies that showing or hiding weekend time can translate a visual layout choice into boundary-relevant placement and verification decisions, motivating our focus on both final placements and attempt-level verification and correction near boundary-adjacent regions [13,15,16].

Weekend time is a salient boundary, not merely another column. Weekends are culturally salient as recovery and leisure time, and weekend work is associated with unequal access to shared family and social time [18]. Weekend recovery is linked to week-to-week fluctuations in performance and affect, suggesting downstream consequences when work moves into weekends [19,20]. Working during non-standard times can also undermine intrinsic motivation and blur boundaries between work and non-work activities [11,21]. Accordingly, weekend visibility can serve as a boundary cue that changes perceived availability and increases verification around boundary-adjacent placements.

Two gaps motivate our study. First, prior work often emphasizes outcomes (e.g., placements, adoption, or self-report) while the micro-process of “getting there”—searching, evaluating, repairing, and confirming candidate placements—is either described qualitatively or collapsed into coarse aggregates such as total completion time. Second, calendar-based boundary interventions (e.g., protecting focus time or discouraging non-work scheduling) frequently evaluate policy-level outcomes, leaving how specific interface representations translate into boundary decisions through concrete cognitive pathways (e.g., option salience/availability and norm signaling) under-specified. We address these gaps by pairing an attempt-centric decomposition of scheduling episodes with event-aligned eye-tracking measures that capture when and where verification and correction occur.

We therefore adopt an attempt-centric, process-oriented view of scheduling. We decompose each scheduling episode into a sequence of attempts—discrete placement actions that may be accepted or revised—and use AttemptsCount (the number of attempts required to reach a final placement) as an objective friction outcome. We treat AttemptsCount as an interaction-cost and correction-cost measure rather than a direct proxy for latent “constraint pressure,” consistent with standards-oriented views of efficiency that include correction work [22,23,24].

We connect attempt-based outcomes to process mechanisms using eye tracking integrated with interaction logs. Eye tracking is a long-standing usability and process diagnostic tool [25,26,27]. Event-aligned approaches segment gaze relative to interaction events and support phase-based interpretations of evaluation and verification [28,29]. Drawing on decision-process accounts that link attention to verification and choice construction, we interpret gaze patterns as evidence of when users search, evaluate, and confirm placements [14,30].

Empirically, we report two experiments with realistic scheduling interfaces. Experiment 1 uses a drag-and-drop interaction grammar aligned with direct manipulation [31] and documents motor-control trade-offs of dragging [32,33]. Experiment 2 uses a dropdown-based time-selection grammar common in scheduling widgets and interactive menus [34,35] and manipulates horizon cues by either making the weekend visible or hiding the weekend while expanding weekday boundary time (e.g., evenings).

Our analyses address five research questions: (RQ1) how interaction grammar (drag vs. dropdown), horizon cues (weekend visible vs. hidden within Experiment 2), and task type relate to attempt-based friction (AttemptsCount); (RQ2) whether AttemptsCount predicts gaze-based process cost, supporting its construct validity; (RQ3) how within-attempt attention shifts across Early/Mid/Late phases and whether phase trajectories differ by horizon within the dropdown interface; (RQ4) whether personal versus work tasks differ in Late-phase weekend verification when the weekend is visible; and (RQ5) whether horizon cues and task type shape boundary outcomes such as weekend placement versus displacement into weekday evenings. These questions connect horizon cues to both process mechanisms (verification dynamics) and boundary outcomes (placement displacement).

The paper contributes (1) a framing of calendar horizon as a boundary affordance grounded in chrono-design and boundary-management perspectives [8,11], (2) an auditable attempt-centric friction measure paired with event-aligned gaze analysis [22,29], and (3) empirical evidence that iterative attempts are common, that AttemptsCount covaries with gaze-based process costs, and that horizon design can reshape boundary-relevant checking and displacement patterns even when its effect on retries is not uniform across tasks.

## 2. Literature Review

### 2.1. Calendars as Socio-Technical Scheduling Systems

CSCW scholarship emphasizes that calendaring is not a purely individual productivity practice; it is a socio-technical activity shaped by organizational routines, shared norms, and coordination demands [1,2,4]. Grudin’s case study highlighted adoption frictions, incentive misalignments, and the importance of organizational context for understanding calendar behavior [1]. Palen similarly framed calendars as social artifacts in which scheduling practices reflect both personal preferences and broader coordination needs [2]. Related work has shown how calendaring practices intertwine with email and communication workflows, and how users manage ambiguity, visibility, and accountability through calendar artifacts [3,4].

As calendars became more central, researchers designed systems to reduce scheduling burdens through automation and mediation [36,37]. Bank and colleagues (2012) [36] proposed turning personal calendars into scheduling assistants that recommend or negotiate options on a user’s behalf. Calendar.help extended this idea by combining automation with human-in-the-loop workflows, emphasizing that scheduling remains delicate even when partially delegated [37]. These systems highlight a recurring tension: reducing coordination work can simultaneously increase the need for verification and correction when the system’s choices do not match user intent or norms [38].

Calendar interaction is also shaped by how time is represented [6,7]. Research on visual time representations suggests that time is not perceived uniformly; structural properties of representations (partitions, overlays, density) influence interpretation, memory, and decision making [6,7]. Chrono-design work argues that time representations embed values and norms, shaping what users treat as “normal” or “acceptable” temporal behavior [8]. These perspectives motivate treating calendar horizons as not only navigation devices, but also normative cues about what time is “available.”

Importantly, the socio-technical view implies that “calendar work” is not only reflected in final placements, but also in the micro-process by which users generate, evaluate, and revise candidate times [1,2,4,5]. In practice, scheduling often unfolds as iterative trial-and-repair: users test a placement, check fit against commitments and norms, and revise when a candidate slot feels infeasible or inappropriate [2,4]. This motivates process-oriented measurements that capture revision structure (e.g., attempt sequences) rather than treating scheduling as a single-shot selection problem [36,37].

### 2.2. Interaction Grammar and the Cost of “Trying”

A second study concerns how interaction grammar structures the cost and style of exploration [31]. Direct manipulation emphasizes continuous, incremental control and immediate feedback, making “try-and-adjust” interactions comparatively cheap [31]. In contrast, menu-based or form-based selection often concentrates decisions into discrete commitment points, which can shift user effort toward post-selection verification and correction. These differences matter for scheduling because users routinely test candidate placements before committing, and interaction grammar can change (i) how often users revise (attempt frequency) and (ii) when verification tends to occur within an attempt (e.g., earlier scanning versus Late-stage confirmation). Work on interactive menus provides complementary insight into dropdown-based scheduling. Byrne et al. examined cognitive and interaction factors in menu selection, showing that selection methods affect search and decision processes [34]. Bailly et al. modeled menu selection as a sequence of micro-strategies, emphasizing that users adapt strategies based on interface structure and costs [35]. From a cognitive perspective, interaction can also function as epistemic action: external actions (e.g., moving or placing an object) can reduce internal computation and support problem solving [39]. Together, these accounts suggest that interaction grammar can shape the prevalence of retries and the distribution of attention during scheduling. Accordingly, an attempt-based friction metric (AttemptsCount) and phase-based gaze allocation provide a principled way to operationalize how different grammars redistribute “trying” and “checking” in scheduling episodes.

### 2.3. Calendar Horizon as Boundary Affordance

Boundary-management theory conceptualizes boundaries between roles (e.g., work and non-work) as actively constructed and maintained through practices, routines, and artifacts, with individuals varying in their preferred degree of segmentation versus integration [9,10,11,12]. Digital calendars are a central boundary artifact because they operationalize commitments into visible temporal structure: what is shown, shaded, or omitted can shape what users treat as legitimate “candidate time” for scheduling and how carefully they inspect boundary-adjacent options.

We conceptualize the calendar horizon—the temporal scope rendered as the default scheduling surface—as a boundary affordance. Horizon cues can influence boundary decisions through at least two complementary cognitive pathways. First, visibility alters the experienced choice set via availability and salience: options that are on-screen are easier to retrieve, compare, and justify, consistent with availability-based judgment and attention-driven choice construction [13,14]. Second, horizon defaults can function as soft normative signals that communicate what is typical or appropriate (e.g., a “workweek” view implying weekday time as the default). Such norm signaling aligns with work on descriptive/injunctive norms and with digital nudging perspectives, where interface defaults and presentation steer behavior by shaping perceived standards and expectations [15,17]. In calendar settings, making weekend time visible may lower the psychological threshold for considering weekend placements, whereas hiding weekend time may raise the threshold while potentially shifting scheduling pressure toward other boundary-adjacent periods [15,17].

Weekend time is a particularly salient boundary because it is socially patterned as recovery and shared leisure time, and weekend work reduces access to shared family and social time [18]. Occupational health research links weekend recovery to subsequent performance and affect, suggesting downstream consequences when work expands into weekends [19,20]. Importantly, boundary interventions that de-emphasize weekends may not eliminate boundary pressure; they may displace it to adjacent “edge” times such as weekday evenings, which can also undermine autonomy and blur work–non-work boundaries [11,21]. This motivates analyses that examine both outcomes (weekend vs. evening placements) and process mechanisms (verification and correction behavior) by which horizon cues shape boundary enactment.

### 2.4. AttemptsCount as a Standards-Aligned Friction Metric

Usability standards define usability in terms of effectiveness, efficiency, and satisfaction [22]. In interactive tasks, efficiency can be operationalized not only as time, but also as the amount of interaction required to complete the task, including errors and correction work [22,23,40]. HCI work similarly argues for objective usability measurements when comparing designs [23,24,40]. Within this framing, an attempt-based metric can capture correction structure directly: each additional attempt reflects incremental effort in revising, verifying, or recovering from an initial placement. Within this framing, AttemptsCount operationalizes observable correction and revision work: each additional attempt reflects a discrete cycle of proposing, checking, and repairing a placement rather than merely “taking longer.”

Attempts become more interpretable when linked to attention measures. Interaction traces can reveal hidden effort and help predict user experience [41]. In decision tasks, attention allocation is tightly linked to choice, and gaze can reflect preference construction and verification processes [14,30]. These perspectives motivate pairing AttemptsCount with gaze-based process measures rather than treating it as a standalone signal. Recent UX research also cautions that efficiency metrics should be interpreted in relation to task structure and user intent, emphasizing that observable interaction costs gain meaning only when grounded in the specific activity being supported rather than treated as abstract performance indicators [42]. In this study, we therefore test construct validity directly by examining whether AttemptsCount covaries with gaze-based process cost (total fixation duration), consistent with the interpretation that additional attempts reflect additional search and verification effort rather than arbitrary interaction noise.

### 2.5. Eye-Tracking and Event-Aligned Process Analysis for Interactive Tasks

Eye tracking is widely used in HCI and usability research to infer attention distribution, cognitive effort, and task difficulty [25,26,27]. Studies show that gaze measures can discriminate task difficulty when paired with subjective and behavioral measures [43] and can capture workload differences in interactive contexts [44]. However, aggregate gaze metrics often erase temporal structure and strategy [45,46,47,48]. Scanpath and visual analytics approaches emphasize that gaze sequences can reveal strategies and phases not visible in totals [45,46,47,48]. Visual analytics research similarly argues that combining interaction data with temporally structured eye-movement analysis is critical for understanding complex user behavior, as aggregate gaze measures alone obscure strategy shifts and decision phases [49].

Event-aligned approaches address this limitation by segmenting gaze relative to interaction events. Sendhilnathan et al. propose an event-aligned approach that segments gaze relative to task phases and transition points, enabling process interpretations beyond static AOI totals [29]. Prior work also demonstrates that eye-movement patterns can reveal confusion, expertise differences, and momentary cognitive states during interactive tasks, particularly when gaze is analyzed alongside behavioral traces rather than in isolation [50,51]. Similar event-based methods align gaze to decision phases and reveal verification and evaluation dynamics [14,28]. AOI definition is also consequential: structural AOIs provide a conservative basis for comparison across designs, while dynamic AOIs can increase precision for moving objects but require careful implementation [52]. Beyond dedicated screen-based eye trackers, recent computer-vision research has advanced fine-grained gaze estimation and head-pose-based attention recognition using monocular RGB data, which may enable more ecologically valid, in-the-wild attention measurement when instrumented lab setups are impractical. Examples include ADGaze for fine-grained gaze estimation via anisotropic label-distribution learning [53] and distribution-learning approaches for head pose estimation and attention recognition such as DADL [54], MFDNet [55], and CNN-based head pose estimation on HPD5A for attention recognition in HCI settings [56]. While our study uses a screen-based Tobii tracker to maximize measurement fidelity and auditability for event-aligned analyses, these methods point to a feasible pathway for future studies to test calendar horizon effects under more naturalistic workplace conditions. These methodological considerations motivate an approach that combines interaction logging with event-aligned gaze analysis over stable, interface structure AOIs.

## 3. Materials and Methods

### 3.1. Study Overview and Design Rationale

Calendar scheduling is an iterative coordination activity rather than a one-shot selection: people evaluate constraints, try candidate placements, revise, and verify fit against other commitments and boundaries (e.g., [1,2,3]). Prior scheduling-assistant work similarly suggests that automation rarely removes the need for users to inspect, repair, and negotiate placements, especially when constraints and preferences are only partially visible or socially situated [36,37].

To capture this iterative structure, we use an attempt-centric approach. Each task scheduling episode is decomposed into a sequence of discrete attempts (placement actions that may be accepted or revised). Our primary behavioral outcome is the number of attempts required to reach a final placement, treating additional attempts as observable correction/revision work.

We report two experiments that vary (a) interaction grammar and (b) horizon cues:

- Experiment 1 (EXP1; drag interface): direct manipulation scheduling using drag and drop.
- Experiment 2 (EXP2; dropdown interface): menu selection scheduling using dropdown choices; within EXP2, calendar horizon is manipulated by whether the weekend region is visible in the view (weekend visible vs. weekend hidden).

Because EXP1 and EXP2 differ in both interface grammar and other study specifics (e.g., task sets), causal inference about horizon is focused on the within-EXP2 contrast (weekend visible vs. weekend hidden). Cross-experiment comparisons that involve EXP1 are treated as convergent/descriptive evidence about interaction techniques. In addition, the task battery differs between EXP1 and EXP2 (see Section 3.4 and the task list in Appendix A), so cross-experiment contrasts are treated as descriptive rather than causal.

### 3.2. Participants

Participants were master’s-level students recruited from a local university and received a gift voucher for participation. Inclusion criteria were normal or corrected-to-normal vision and no self-reported serious eye conditions.

A total of 131 participants were recruited (EXP1: *n* = 52; EXP2A: *n* = 42; EXP2B: *n* = 37). We excluded 26 participants based on pre-specified eye-tracking data-quality criteria (Section 3.7), yielding a final sample of 105 retained participants (EXP1: *n* = 34; EXP2A: *n* = 41; EXP2B: *n* = 30). Participant demographics by experiment (age and self-reported sex/gender) are reported in Table 1.

### 3.3. Apparatus and Eye-Tracking Procedure

Eye movements were recorded using a screen-based Tobii Pro Nano eye tracker (Tobii AB, Danderyd, Sweden; sampling rate: 60 Hz). Stimuli were presented on a 15.6-inch laptop screen (VivoBook Pro 15, model M6500X; ASUSTeK Computer Inc., Taipei, Taiwan) in 1920 ∗ 1080, with an approximate viewing distance of 60 cm. A 9-point calibration was performed at the start of the session (repeated if validation failed). If calibration/validation did not meet the criteria (less than 1.0) after repeated attempts, the session was terminated and the participant was excluded from analysis. Tobii Pro Lab (version 1.23; Danderyd, Sweden: Tobii AB) was used to define AOIs and export fixation measures.

### 3.4. Tasks, Interfaces, and Experimental Conditions

Participants scheduled a series of work and personal tasks into a prepopulated weekly calendar with occupied timeslots. Tasks were labeled as work or personal to test whether boundary-relevant tasks elicit different revision and verification patterns. The task battery consisted of realistic items displayed in the task panel. EXP1 included 8 work tasks and 6 personal tasks; EXP2 included 8 work tasks and 3 personal tasks (full task list provided in Appendix A).

Task type (work vs. personal) was assigned a priori; the final analytic dataset contained only these two categories (no “other” category after cleaning).

EXP1 (drag): Participants scheduled by dragging task items into timeslots.

EXP2 (dropdown): Participants scheduled using dropdown selection embedded in the calendar grid. Horizons were manipulated as follows:

- Weekend visible (EXP2A): a weekend region was present in the view.
- Weekend hidden (EXP2B): the weekend region was not present in the view.

**Figure 1:**
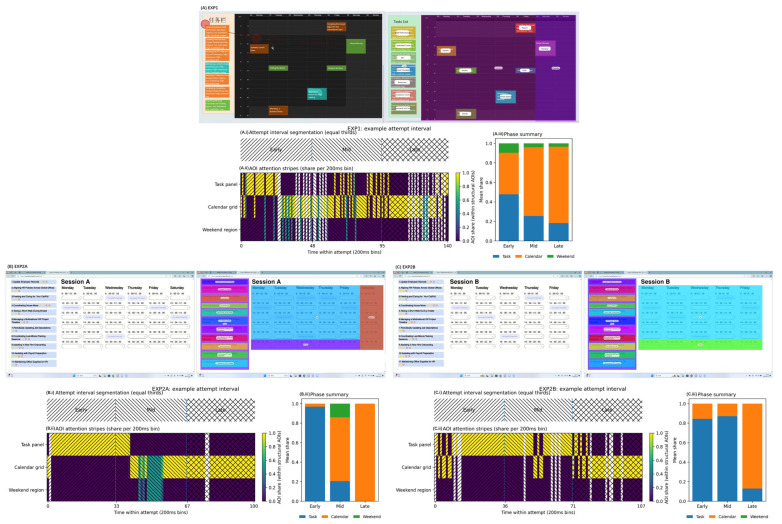
Interfaces/conditions and the attempt-centric, event-aligned gaze pipeline. (**A**) EXP1 (drag), (**B**) EXP2A (dropdown + weekend visible), and (**C**) EXP2B (dropdown + weekend hidden). Structural AOIs include the task panel, calendar grid, and (when present) weekend region; each attempt interval is partitioned into Early/Mid/Late phases via equal-third segmentation.

Evening time was available in EXP2; placement coding (below) captures whether final placements landed in evening slots. Figure 1 provides representative interfaces for each condition and summarizes the structural AOIs and within-attempt phase segmentation used in the gaze analysis pipeline.

### 3.5. AOIs and Event-Aligned Segmentation

#### 3.5.1. Structural AOIs

To preserve auditability and reduce sensitivity to dynamic AOI drift, we used structural AOIs corresponding to stable interface regions:

- Task panel (task list/source area)
- Calendar grid (main scheduling surface)
- Weekend region (only when present in the interface)

We intentionally used coarse, structural AOIs to maintain auditability and comparability across interface variants and to minimize dynamic AOI drift. As a result, we did not interpret gaze at the level of individual grid cells or specific conflict markers in the analyses.

#### 3.5.2. Task Episodes, Attempt Intervals, and Phases

We aligned gaze to interaction structure using a nested segmentation:

- A task episode (TOI) spans a participant’s work on one scheduling task.
- Within a TOI, an attempt interval corresponds to one distinct placement attempt.
- Each attempt interval is partitioned into Early/Mid/Late phases based on relative time within that interval (equal-third segmentation).

This phase framing supports a conservative interpretation of Late-phase attention as “verification proximal” (i.e., attention allocation in the closing portion of an attempt, when users are plausibly confirming fit).

Attempt intervals were identified from the interaction logs within each task episode: each recorded placement action for a given task was treated as one attempt, and successive attempts were delimited by consecutive placement actions until the final placement was reached. Because attempt durations vary substantially, we used equal-third segmentation to normalize each attempt and to capture the transition from initial orientation/search (Early), through candidate evaluation (Mid), to verification proximal processing near commitment (Late).

### 3.6. Measures

Notation. Throughout, i indexes participants, t indexes task episodes (participant × task), m indexes attempt intervals within a task episode, ph∈{Early,Mid,Late} indexes within-attempt phases, a indexes AOIs, $A_{\mathrm{struct}}$ denotes the set of structural AOIs, and $I_{i,t}$ denotes the set of attempt intervals for task episode (i,t).

#### 3.6.1. Attempt-Based Friction (Task-Level)

We operationalize attempt-based friction as the number of discrete placement attempts a participant makes before reaching a final placement within a task episode. Let participant i work on task episode t, and let $I_{i,t}$ denote the set of attempt intervals identified within that task episode. We define the following:

(1) $${AttemptsCount}_{i,t}=|I_{i,t}|$$

${AttemptsCount}_{i,t}$ is the primary behavioral friction outcome. Let $A_{struct}$ be the set of structural AOIs (task panel, calendar grid, and—when present—the weekend region). Let ${FixDur}_{i,t,a}$ be the total fixation duration (ms) in AOI a during task episode(*i*,*t*). We compute the following:

(2) $${FixDurStruct}_{i,t}=\sum_{a\in A_{struct}}{FixDur}_{i,t,a}$$

Because fixation durations are right-skewed, models use the natural-log-transformed outcome $log({FixDurStruct}_{i,t})$. For phase-based allocation, let $F_{i,t,m,ph,a}$ denote fixation duration (ms) in AOI a within attempt interval m and phase ph. Structural fixation within that unit is as follows:

(3) $$F_{i,t,m,ph,struct}=\sum_{a\in A_{struct}}F_{i,t,m,ph,a}$$

AOI share is defined as follows:

(4) $$S_{i,t,m,ph,a}=\frac{F_{i,t,m,ph,a}}{F_{i,t,m,ph,struct}}(\mathrm{defined}\;\mathrm{when}\;F_{i,t,m,ph,struct}>0)$$

Key indices include the following:

- CalendarShare: $S_{i,t,m,ph,\mathrm{calendar}}$
- WeekendShare: $S_{i,t,m,ph,\mathrm{weekend}}$ (only when weekend AOI exists)
- LateWeekendShare: WeekendShare computed in the Late phase of each attempt interval

We interpret Late-phase attention as verification proximal because it reflects attention allocation near the end of the attempt interval. Evening slots were defined as time slots after 18:00 in the EXP2 calendar grid (i.e., outside standard daytime working hours in our interface implementation).

#### 3.6.2. Boundary Placement Outcomes (Task-Level)

To capture boundary-relevant outcomes, final placements are coded from the end state of each task episode:

- PlacedWeekend: ${PlacedWeekend}_{i,t}=1\;$ if the final placement lands on the weekend region (defined only when the weekend region is available/visible). In our interfaces, “weekend” is operationalized via the weekend column shown in the view (treating Saturday as the weekend proxy where applicable).
- PlacedEvening: ${PlacedEvening}_{i,t}=1\;$ if the final placement lands in an evening slot (defined in EXP2 where evening time is available).

### 3.7. Data Export, Preprocessing, and Quality Control

Fixations were exported from the eye-tracking software in 200 ms bins and aligned to the nested segmentation described above (task episode → attempt intervals → Early/Mid/Late phases). Recordings were excluded prior to analysis if they failed pre-specified eye-tracking data-quality thresholds, including (i) a valid gaze sample ratio below 75% over the task period and/or (ii) continuous tracking dropout exceeding 15 s during task execution.

For AOI share computations, units with zero structural fixation were treated as missing to avoid undefined ratios:

If $F_{i,t,m,ph,\mathrm{struct}}=0$, then $S_{i,t,m,ph,a}$ is set to missing for that unit.

Very short or malformed intervals were retained for AttemptsCount when they corresponded to genuine revisions (i.e., they represent real attempts in the interaction stream). However, if an interval was too short to support meaningful Early/Mid/Late partitioning, it could be excluded from the phase-decomposition summaries while still contributing to AttemptsCount.

**Figure 2:**
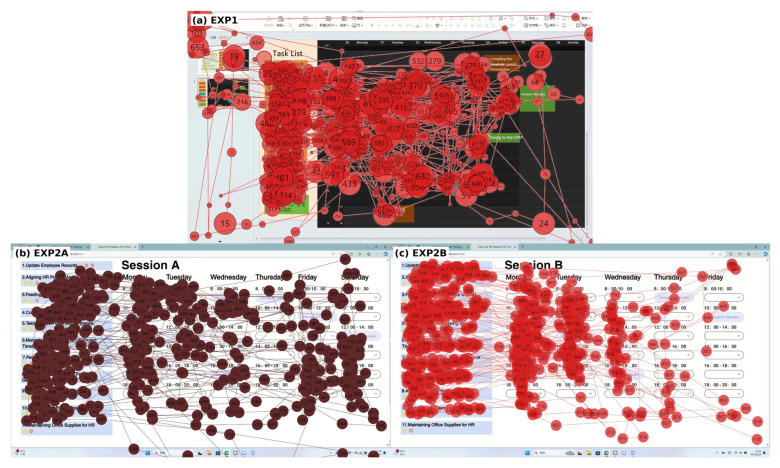
Representative scanpaths. (**a**) EXP1 (drag), (**b**) EXP2A (dropdown + weekend visible), (**c**) EXP2B (dropdown + weekend hidden). Circles indicate fixations (size proportional to duration) and lines indicate saccades.

Representative scanpaths are provided in Figure 2 to illustrate attention distribution patterns across interface conditions (fixations shown as circles scaled by duration, saccades as connecting lines).

### 3.8. Statistical Analysis

All inferential analyses use participant-clustered robust standard errors to account for repeated task observations within participants. We report effect sizes in interpretable forms (IRRs for count models; odds ratios for logistic models) with corresponding confidence intervals. All statistical tests were two-sided. We report exact *p*-values when possible and report p<0.001  when *p*-values are smaller than 0.001.

#### 3.8.1. AttemptsCount (Count Outcome)

We model ${AttemptsCount}_{i,t}\;$ using Poisson GLMs with a log link and report incidence rate ratios (IRR). A generic specification is as follows:

(5) $$log\left(E[{AttemptsCount}_{i,t}]\right)=\beta_{0}+\beta_{1}{Personal}_{i,t}+\beta_{2}{Drag}_{i,t}+\beta_{3}{WeekendVisible}_{i,t}$$

Here, ${Personal}_{i,t}\in \left\{0,1\right\}\;$ indicates task type (1 = personal, 0 = work). ${Drag}_{i,t}\in \left\{0,1\right\}$ indicates the drag-and-drop interface condition (EXP1). ${WeekendVisible}_{i,t}\in \left\{0,1\right\}\;$ indicates whether the weekend region is present in the calendar view (within EXP2). We report $IRR=exp(\beta_{k})$.

#### 3.8.2. Construct Validity: Gaze Cost as a Function of Attempts

To test whether behavioral friction covaries with gaze-based process cost, we model log fixation duration using OLS:

(6) $$log({FixDurStruct}_{i,t})=\gamma_{0}+\gamma_{1}{AttemptsCount}_{i,t}+\gamma_{2}{Personal}_{i,t}+\delta_{EXP(t)}+\varepsilon_{i,t}$$

$\gamma_{EXP}\;$ denotes experiment fixed effects (as applicable). The quantity $\exp\left(\gamma_{1}\right)\;$ is interpreted as the multiplicative change in fixation duration associated with one additional attempt.

#### 3.8.3. Placement Outcomes (Binary)

Boundary placement outcomes are analyzed using logistic GLMs with participant-clustered robust standard errors, as illustrated by the model for evening placement:

(7) $$logit\left(Pr({PlacedEvening}_{i,t}=1)\right)=\alpha_{0}+\alpha_{1}{Personal}_{i,t}+\alpha_{2}{WeekendVisible}_{i,t}$$

We report odds ratios $exp(\alpha_{k})$. Models for PlacedWeekend are estimated only on observations where the weekend region is available/visible (so that PlacedWeekend is defined).

### 3.9. Research Questions

We address five research questions:

- RQ1 (friction): How do interaction grammar (drag vs. dropdown), horizon (weekend visible vs. hidden), and task type (personal vs. work) affect attempt-based friction (AttemptsCount)?
- RQ2 (construct validity): Does AttemptsCount predict gaze-based process cost (structural fixation duration)?
- RQ3 (within-attempt dynamics): How does attention to the calendar surface shift across Early/Mid/Late phases, and does this differ by horizon within EXP2?
- RQ4 (weekend verification): When the weekend is visible, do personal tasks show greater Late-phase weekend attention than work tasks?
- RQ5 (boundary outcomes): Do horizon cues and task type shift where tasks end up (weekend vs. evening placements)?

## 4. Results

### 4.1. Analytic Sample and Descriptive Overview

After preprocessing and exclusion based on eye-tracking quality criteria, the analytic dataset comprised 1018 task episodes contributed by 105 participants across three conditions: EXP1 (drag and weekend visible; 337 tasks from 34 participants), EXP2A (dropdown and weekend visible; 402 tasks from 41 participants), and EXP2B (dropdown and weekend hidden; 279 tasks from 30 participants). Unless otherwise noted, results are reported at the task episode level, with inference based on participant-clustered standard errors to account for repeated observations within participants.

**Figure 3:**
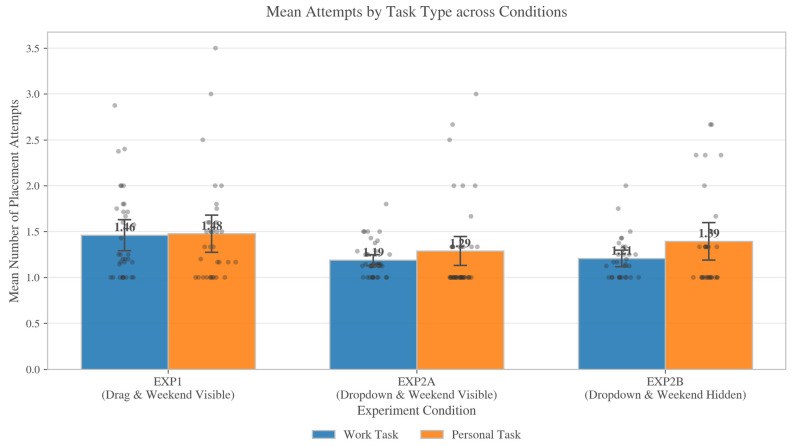
Mean number of placement attempts (AttemptsCount) by task type (work vs. personal) across conditions. Gray points denote individual task episodes, bars show condition means, and error bars indicate uncertainty around the mean (see analysis script, e.g., ±1 SE). Task episode counts were EXP1 = 337, EXP2A = 402, and EXP2B = 279.

Across conditions, AttemptsCount ranged from 1 to 7, indicating that iterative “try–revise” behavior was common but typically bounded. The distribution was strongly skewed toward single-attempt completion, though the prevalence differed by condition: 66.2% of tasks were completed in one attempt in EXP1 (M = 1.47, SD = 0.83), compared with 82.8% in EXP2A (M = 1.20, SD = 0.50) and 80.3% in EXP2B (M = 1.27, SD = 0.63). Figure 3 provides descriptive context by showing AttemptsCount by task type across conditions.

Task-level gaze-based process cost (total fixation duration within structural AOIs) was variable, with mean durations on the order of 14–16 s: EXP1 mean = 16,419 ms (median = 12,301 ms), EXP2A mean = 13,861 ms (median = 9997 ms), and EXP2B mean = 14,738 ms (median = 11,080 ms). These descriptive patterns provide context for the inferential analyses reported below.

### 4.2. RQ1: Interface, Horizon, and Task-Type Effects on AttemptsCount

RQ1 examined whether attempt-based friction varied with interaction grammar (drag vs. dropdown), horizon cues (weekend visible vs. hidden), and task type (personal vs. work).

In the pooled Poisson model with participant-clustered standard errors, drag was associated with a higher attempt rate than dropdown (IRR = 1.219, 95% CI [1.086, 1.368], *p* < 0.001), corresponding to approximately 22% more placement attempts under drag-and-drop scheduling. Because this grammar contrast is observed across two experiments that also differ in task batteries and other implementation details, we treat it as convergent/descriptive evidence of an interface grammar difference rather than an isolated causal estimate; causal inference about horizon is therefore focused on the within-EXP2 comparison below.

Within EXP2 (dropdown only), weekend visibility was not a reliable predictor of AttemptsCount (IRR = 0.944, 95% CI [0.859, 1.038], *p* = 0.237). By contrast, task type showed a consistent effect: across EXP2, personal tasks required more attempts than work tasks (IRR = 1.103, 95% CI [1.014, 1.200], *p* = 0.022). This personal-task attempt penalty was larger when the weekend was hidden (EXP2B only: IRR = 1.156, 95% CI [1.002, 1.335], *p* = 0.047), suggesting that constraining the visible candidate space disproportionately increases revision effort for personal scheduling. Figure 3 summarizes these patterns descriptively.

### 4.3. RQ2: Construct Validity—AttemptsCount Predicts Gaze Cost

**Figure 4:**
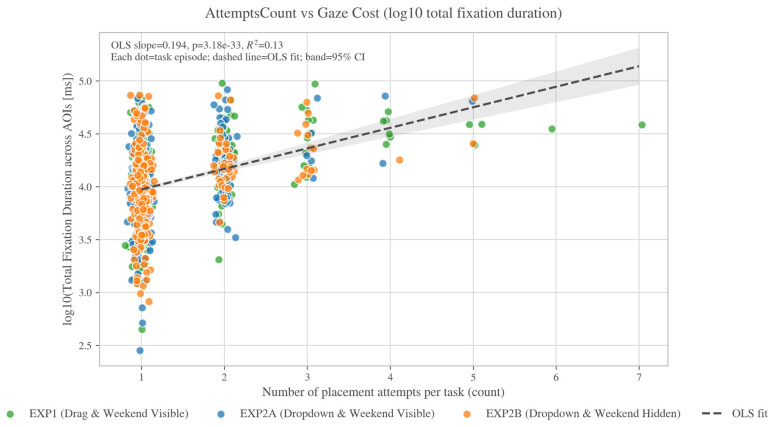
Association between attempt-based friction and gaze-based process cost. Each point represents one task episode (colored by condition). The y-axis plots total fixation duration within structural AOIs on a log10 scale for visualization. The grey dashed line indicates the OLS linear fit, and the grey shaded area shows the 95% confidence interval around the fitted trend, illustrating that higher AttemptsCount is associated with higher gaze cost.

RQ2 tested the construct validity of AttemptsCount by examining whether additional attempts were associated with higher gaze-based process cost. In the task-level model predicting log structural fixation duration (with experiment fixed effects and participant-clustered standard errors), AttemptsCount was a strong positive predictor of gaze cost (β = 0.443, *p* < 0.001). This corresponds to a multiplicative increase in exp(β) = 1.557 (95% CI [1.417, 1.710]), i.e., an estimated ~56% increase in total structural-AOI fixation duration for each additional attempt, holding task type and experiment constant. This association supports interpreting AttemptsCount as capturing substantive processing effort (search, verification, and correction) rather than a purely mechanical interaction count. Figure 4 visualizes this monotonic relationship.

### 4.4. RQ3: Within-Attempt Attention Dynamics (Attempt × Phase Analysis)

RQ3 examined whether attention allocation within an attempt changed systematically across Early/Mid/Late phases, and whether those dynamics differed by horizon within EXP2. Using the 200 ms binned exports aggregated to attempt × phase, CalendarShare increased toward the Late phase in both EXP2 conditions, consistent with a “place then verify” sequence. Mean CalendarShare progressed from 0.443 → 0.491 → 0.635 across Early/Mid/Late in EXP2A (weekend visible), and from 0.501 → 0.467 → 0.664 in EXP2B (weekend hidden).

**Figure 5:**
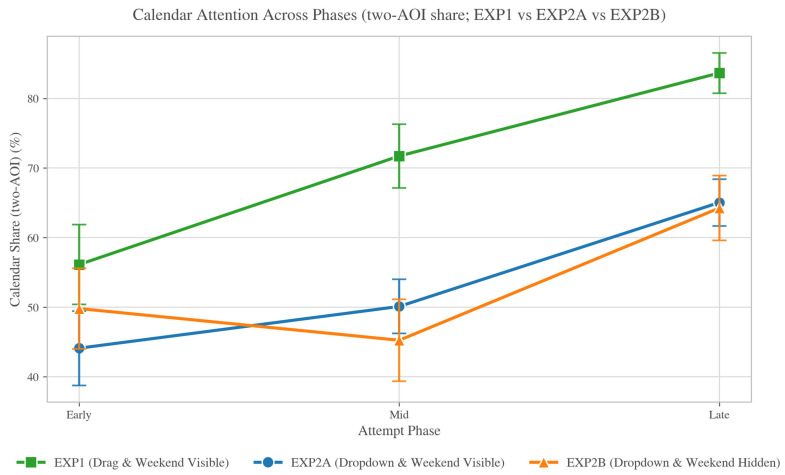
CalendarShare across attempt phases (Early/Mid/Late) by condition. Points show mean CalendarShare within each phase (aggregated over attempt × phase observations), with error bars indicating uncertainty around the mean (e.g., ±1 SE). CalendarShare increases toward the Late phase in both EXP2 conditions, consistent with end-of-attempt verification on the calendar surface.

Two features are noteworthy. First, Late-phase calendar dominance was robust: attention concentrated on the calendar surface near attempt completion, consistent with end-of-attempt confirmation. Second, when the weekend was hidden, calendar attention was more front-loaded (higher Early CalendarShare), consistent with more upfront scanning when the visible horizon is compressed, while still converging on high Late-phase checking. Figure 5 summarizes these phase trajectories; EXP1 is included for descriptive context, whereas horizon-related comparisons are interpreted within EXP2A vs. EXP2B.

### 4.5. RQ4: Late-Phase Weekend Verification by Task Type

RQ4 assessed whether personal versus work tasks differ in boundary-relevant verification when the weekend region is visible. We focused on LateWeekendShare as a conservative proxy for weekend checking near attempt completion, and we evaluated this primarily within EXP2A (weekend visible) to preserve within-experiment inference.

**Figure 6:**
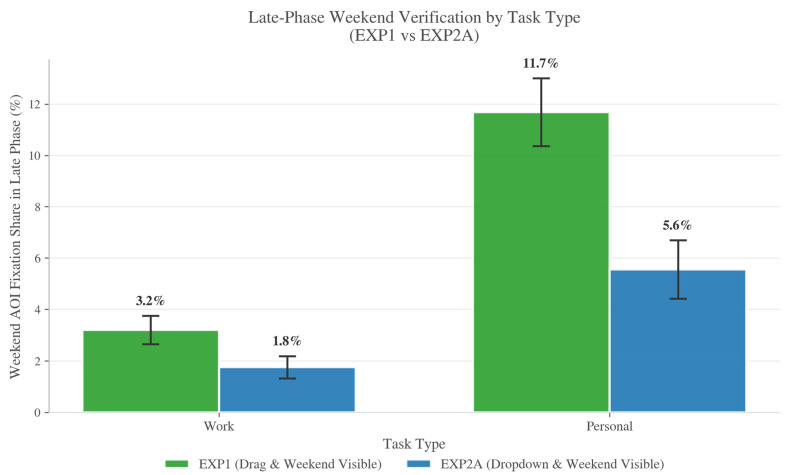
Late-phase weekend verification (LateWeekendShare) by task type under weekend-visible conditions (EXP1 vs. EXP2A). Bars show participant-level means of LateWeekendShare; error bars indicate uncertainty across participants (e.g., ±1 SE). Personal tasks show higher Late-phase weekend attention than work tasks in EXP2A (*n* = 41), consistent with stronger boundary-oriented checking near attempt completion.

In EXP2A, personal tasks elicited greater Late-phase weekend attention than work tasks (participant-level means: 5.6% vs. 1.8%, *n* = 41). The within-participant personal–work contrast was statistically reliable (paired t(40) = 2.13, *p* = 0.040; Wilcoxon signed-rank *p* = 0.037). This pattern is consistent with stronger end-of-attempt checking of the weekend boundary before committing to personal placements. Figure 6 reports LateWeekendShare by task type; EXP1 shows a similar directional pattern descriptively.

### 4.6. RQ5: Boundary Placement Outcomes

RQ5 examined boundary-relevant end states by coding each task’s final placement, focusing on weekend placement (defined only when the weekend region is visible) and evening placement (defined in EXP2 where evening time is available).

For weekend placement within EXP2A (weekend visible; N = 402 tasks), personal tasks were more likely than work tasks to end on the weekend (personal: 16.2% of 117 tasks; work: 5.3% of 285 tasks). A clustered logistic model estimated substantially higher odds of weekend placement for personal tasks (OR = 3.49, 95% CI [1.48, 8.22], *p* = 0.0043). EXP1 exhibited a similar directional difference (personal 39.1% vs. work 5.3%), but EXP1 was not used to identify the causal impact of horizon cues.

For evening placement in EXP2 (weekend visible vs. hidden; N = 681 tasks), evening placement occurred in both horizon conditions but was consistently more common for personal tasks. In EXP2A (weekend visible), evening placement was 32.5% for personal tasks versus 3.5% for work tasks; in EXP2B (weekend hidden), it increased to 42.0% for personal tasks versus 8.6% for work tasks. In a clustered logistic model pooling EXP2, personal tasks had markedly higher odds of evening placement (OR = 9.93, 95% CI [5.37, 18.38], *p* < 0.001). Importantly, weekend visibility reduced the odds of evening placement (OR = 0.55, 95% CI [0.35, 0.87], *p* = 0.011), consistent with the weekend region acting as an additional candidate space that reduces reliance on weekday-evening “boundary expansion”.

Taken together, AttemptsCount captured meaningful interaction friction: it varied with interface grammar and task type, and it covaried strongly with gaze-based process cost. Horizon cues (weekend visible vs. hidden) had a weaker association with retries but systematically shifted where attention was allocated near attempt completion and where tasks ultimately landed (weekend vs. weekday evenings), indicating that horizon design can change boundary outcomes even when attempt counts remain similar.

## 5. Design Implications

Calendars are not neutral displays of time; they function as a choice architecture that shapes which time slots feel salient, permissible, and worth verifying before commitment. In our attempt-aligned analyses, horizon cues (weekend visible vs. hidden) and interaction grammar (drag vs. dropdown) changed not only where tasks were ultimately placed (e.g., weekend vs. weekday evenings) but also when attention concentrated on verification within an attempt [6,7,15,17]. We therefore interpret “calendar horizon” as a boundary-relevant interface affordance: it alters the perceived candidate set and the cognitive work of boundary negotiation through well-established pathways such as salience/availability and norm signaling [10,11,15,17]. The implications below translate these findings into actionable guidance for calendar UI design and, prospectively, for mixed-initiative scheduling tools that increasingly intervene in time allocation [36,37,57,58].

### 5.1. Treat the Calendar Horizon as a User-Facing Boundary Control

Calendar horizons shape boundary decisions because they alter what users treat as the “default” candidate space [15,17]. When weekend time is visually present, it becomes more cognitively available and can be implicitly framed as a legitimate option; when it is hidden, weekend becomes less salient and users may instead search within the visible workweek and extend into adjacent boundary regions such as weekday evenings [11,21]. This is consistent with boundary-management accounts in which boundary strength and permeability are enacted through everyday practices and cues, not only through explicit policies (e.g., segmentation vs. integration preferences and role transitions) [9,10,11,12].

Accordingly, horizon should be treated as an explicit boundary setting rather than a purely navigational preference [17]. Interfaces should do the following:

- make horizon selection explicit and persistent (e.g., workweek vs. full week) and allow one-click switching at the point of placement;
- communicate what is currently out of view (e.g., “weekend hidden”) to avoid false impressions that the weekend is unavailable;
- provide organization- or role-sensitive defaults (e.g., business calendars default to workweek) while allowing user overrides; and
- surface displacement trade-offs (e.g., “hiding the weekend may shift placements toward evenings”) so users understand the boundary consequences of a horizon choice.

### 5.2. Support Boundary Verification Without Forcing Extra Retries

Attempt sequences indicate that scheduling effort is often spent on verification and correction, not merely on initial placement [1,2,36,37]. Even when weekend visibility did not uniformly change AttemptsCount, it shifted when users concentrated attention on the calendar surface—suggesting that interface structure can move verification earlier or later in the attempt.

Design should therefore reduce the cost of boundary-relevant verification without requiring additional attempts. Practical mechanisms include the following:

- pre-commit previews that expose conflicts, duration fit, and boundary-adjacent consequences (e.g., “this placement uses evening time”);
- visual boundary cues that are specific and interpretable (e.g., distinct shading/labels for weekend and evenings) to support deliberate review rather than accidental placement;
- lightweight confirmation affordances only for boundary-crossing actions (e.g., weekend/evening placements) to separate “routine” from “boundary-relevant” commitments; and
- fast undo, stepwise adjustments, and reversible edits so that verification does not escalate into costly multi-step rework [31].

### 5.3. Choose Interaction Grammar Based on the Desired Exploration Style

Interaction grammar changes the “cost of trying.” Drag-and-drop supports continuous, what-if exploration and can encourage incremental trial-and-repair, whereas dropdown slot-picking concentrates decisions into discrete choice points that may encourage users to deliberate before committing [31]. Designers should treat grammar as a policy choice tied to context:

- For planning contexts where exploration is expected (e.g., personal planning), drag can be appropriate, but it should be paired with strong alignment guides, clear boundary cues, and easy reversal to prevent exploratory behavior from becoming error-prone rework.
- For compliance-oriented contexts (e.g., workplace scheduling), constrained selection can reduce accidental placements, but it should not silently remove boundary options that users legitimately need; instead, boundary options should remain accessible with explicit signaling and low-friction verification.

### 5.4. Make Boundary Assumptions Explicit in AI-Assisted Scheduling

While our experiments did not include AI-generated slot recommendations, the present findings still inform mixed-initiative scheduling because both AI and humans operate within the visible “candidate space” defined by the interface horizon [36,37,57,58]. If an assistant recommends times while the weekend is hidden (or highlighted), it inherits the interface’s boundary framing and may unintentionally normalize boundary crossings or displace work into evenings [21]. AI-assisted scheduling should therefore externalize boundary assumptions and make trade-offs inspectable: for example, whether the system is optimizing for earliest availability, minimizing fragmentation, avoiding weekends, or respecting user/organization-defined “protected time” [58,59,60,61]. The interface should present alternative options across boundary categories (weekday daytime vs. evening vs. weekend) and provide low-friction overrides and explanations (“why this slot”) to support calibrated trust and efficient correction when suggestions conflict with user intent [38,59,62].

### 5.5. Avoid “Palliative” Boundary Concealment: Design for Boundary Health

Our placement results suggest a key design risk: hiding the weekend does not necessarily reduce boundary-crossing demand—it may reallocate that demand into other boundary-adjacent regions, notably weekday evenings. From a well-being perspective, this can be a palliative intervention: it improves the appearance of a protected weekend while potentially increasing fragmentation and extending work into late hours [11,18,19,20,21].

Designers should therefore complement horizon controls with explicit boundary-health affordances, such as user-configurable working hours, protected recovery time, and feedback that makes displacement visible (e.g., “this choice increases evening load this week”). In organizational settings, these interface cues can be coupled with policy-aware defaults (e.g., “avoid evenings unless urgent”) while still preserving transparency and user control [17,58,62].

## 6. Discussion

This paper examined calendar horizon as a boundary affordance: a representational decision about what time is visible (e.g., whether weekend appears alongside weekdays) that may reshape both scheduling outcomes and the micro-process of scheduling. Framed through boundary-management theory, horizon cues can be understood as interface-level signals that modulate boundary salience and boundary permeability, thereby shaping how users negotiate role transitions and protect (or trade off) non-work time [10,11,12]. By decomposing scheduling episodes into attempts and aligning gaze to those attempts, we treat scheduling not only as an outcome but as an iterative coordination activity with observable correction and verification work [1,2,36,37].

Across experiments, many tasks were completed in a single attempt, yet multi-attempt episodes were common enough to matter for both usability and boundary outcomes. This supports classic CSCW accounts that treat calendaring as negotiation and repair: observable retries reflect correction and re-evaluation work rather than mere “slowness” [4,5]. In this framing, the key question is not whether people revise—revision is expected—but which interface choices change the frequency, timing, and attentional structure of verification and repair.

Interaction grammar was associated with a different revision structure: the drag interface exhibited higher attempt rates than the dropdown interface, consistent with the idea that continuous manipulation encourages exploratory trial-and-repair while discrete selection concentrates commitment into fewer, more deliberate steps [31]. However, this comparison spans separate experiments with different task sets and contextual details. We therefore interpret grammar effects as convergent evidence rather than as an isolated causal estimate, and we avoid attributing cross-experiment differences to a single design factor.

A central threat to internal validity is that interaction grammar (drag vs. dropdown) is not manipulated within a single balanced design in the present dataset. Because Experiment 1 and Experiment 2 differ in both interface syntax and task settings, cross-experiment contrasts may reflect confounds such as task difficulty, calendar density, instruction framing, or learning effects rather than grammar alone. To isolate grammar and horizon as separable causal factors, future work should adopt balanced experimental designs, such as (i) a 2 × 2 factorial manipulation crossing grammar (drag vs. dropdown) with horizon (weekend visible vs. hidden) under the same task set, or (ii) within-subject counterbalanced designs where each participant completes equivalent tasks under both grammars with horizon held constant. Such designs would enable clearer attribution of observed differences in AttemptsCount and attempt-phase attention dynamics to specific interface variables.

Horizon design, in contrast, manifested most clearly in verification strategy and boundary outcomes rather than in a uniform change in AttemptsCount. Within the dropdown interface (EXP2), weekend visibility did not reliably reduce or increase the number of attempts across tasks; instead, the more consistent signals appeared in attention dynamics and placement displacement patterns. We interpret this through two complementary cognitive pathways. First, visibility changes availability and salience: when the weekend is visually present, it becomes a cognitively accessible option during search, while hiding it reduces the likelihood that users treat it as a default candidate space. Second, visibility can function as a normative cue: presenting the weekend alongside weekdays may signal permissibility (“this is an acceptable place to schedule”), whereas hiding the weekend may signal a workweek norm even though the weekend remains technically available. Together, these pathways provide a concrete bridge between interface representation and boundary-management behavior: horizon cues alter how users construct the feasible choice set, and they modulate the amount and timing of boundary-relevant verification before commitment.

A critical implication of the horizon manipulation is that boundary protection can become displacement rather than prevention [21]. When the weekend region is hidden, tasks that might otherwise have been placed on the weekend do not vanish; instead, they can shift into weekday evenings. This raises a well-being concern: a “weekend-hidden” interface may be palliative if it produces a cleaner-looking workweek view while increasing reliance on late-day scheduling and potentially fragmenting recovery time across the week [18,19,20]. From a boundary-management perspective, such displacement may weaken effective segmentation by extending work into non-standard hours even when the weekend appears protected. Future evaluations should therefore treat “evening expansion” as a first-class boundary outcome alongside weekend placement, and assess downstream consequences such as perceived boundary control, fatigue, and recovery quality.

Boundary work was also task-dependent. When weekend time was visible, personal tasks exhibited stronger Late-phase weekend-oriented verification than work tasks, suggesting that users engaged in additional boundary checking before committing to placements near salient non-work time. Two non-exclusive psychological mechanisms may explain this pattern. One possibility is flexibility-driven exploration: personal tasks may be perceived as more movable, prompting users to test candidate placements and then verify boundary fit at the end of an attempt. A second possibility is normative/affective cost: placing personal commitments near weekend boundaries may carry perceived trade-offs (e.g., protecting downtime or avoiding “giving up” leisure time), leading to increased double-checking and justification prior to commitment [18,19,20]. Our current behavioral and gaze data support the presence of Late-phase verification but cannot adjudicate between these mechanisms; future work should pair attempt-aligned measures with brief self-reports (e.g., perceived guilt, boundary importance, or segmentation preference) or experimentally manipulate boundary norms to test which pathway primarily drives late verification.

A key methodological contribution is evidence that AttemptsCount is not only behaviorally interpretable but also process-grounded. AttemptsCount strongly covaried with gaze-based process cost: additional attempts were associated with substantial increases in total structural-AOI fixation duration. This pattern supports treating AttemptsCount as an efficiency-relevant indicator of iterative correction and verification work, consistent with standards-oriented views that include rework and error recovery as part of interaction cost [22,23,40]. Practically, pairing AttemptsCount with attempt-aligned gaze measures helps distinguish designs that merely shift effort in time (e.g., earlier vs. later checking) from designs that genuinely reduce verification burden.

These results matter for AI-mediated scheduling and calendar governance. Although our experiments did not include AI-generated slot recommendations, mixed-initiative systems will operate within the same representational constraints and boundary cues that shape human scheduling [36,37,57,58]. If horizon cues alter what users treat as an acceptable candidate space and when they perform verification, then AI suggestions that ignore horizon-sensitive boundary assumptions may increase correction work and erode calibrated trust [38,58,62]. A design-relevant implication is that assistants should make boundary policies explicit (e.g., weekend/evening avoidance), show trade-offs, and support low-friction inspection and override (“why this slot?”) so that verification remains efficient and boundary-respecting [57,58,59,62].

Several limitations constrain interpretation and motivate future work. First, grammar effects compare separate experiments and should be interpreted cautiously; balanced designs are needed to isolate grammar and horizon as independent causal factors. Second, our gaze analysis relies on static structural AOIs for robustness and auditability, but this choice limits micro-level inference within the calendar grid (e.g., differential attention to morning vs. evening slots or to fine-grained conflict markers). Future work could introduce sub-regions within the calendar grid or dynamic AOIs to capture within-grid boundary markers while preserving alignment to attempt events [52]. Third, the study was conducted in a controlled setting with a finite set of tasks and a moderate sample size; ecological validity may differ in real-world calendar use where interruptions, organizational norms, and multi-party constraints shape scheduling pressure. Finally, scaling attempt-aligned attention analysis to field contexts may require alternative sensing methods beyond screen-based eye trackers. Recent work on fine-grained gaze estimation and head-pose-based attention recognition (e.g., ADGaze; DADL; MFDNet; and HPD5A-based head pose estimation) suggests promising pathways for in-the-wild measurement that could complement the controlled precision of the present study [55,56].

## 7. Conclusions

Calendar interfaces shape boundary negotiation not only through what users ultimately schedule, but through the micro-process of how they search, verify, and revise candidate placements. By introducing an attempt-centric, gaze-aligned pipeline, we show that AttemptsCount captures meaningful correction work and covaries strongly with attentional process cost. Substantively, weekend visibility functions as a horizon cue that shifts boundary-relevant verification and reallocates placements between weekend and weekday evenings, highlighting the risk that boundary concealment can produce displacement rather than protection. For practitioners—including small and medium-sized enterprises (SMEs) that rely on off-the-shelf calendar tools and have limited administrative capacity—these findings underscore that seemingly minor view defaults (workweek vs. full week) can meaningfully change scheduling friction and boundary outcomes. Designing horizon controls, verification supports, and policy-aware (including AI-assisted) scheduling tools with transparent boundary assumptions can therefore reduce administrative cognitive friction while better supporting sustainable work–life boundaries.

## Figures and Tables

**Table 1:** Participant characteristics and exclusions by experiment.

Experiment	N (Raw)	N (Excluded)	N (Retained)	Age (Mean ± SD)	Age Range	Gender Counts
EXP1	52	18	34	24.63 ± 2.24	23–28	F:26; M:9
EXP2A	42	1	41	24.24 ± 2.96	21–35	F:31; M:10
EXP2B	37	7	30	23.60 ± 1.52	22–27	F:26; M:4

All participants provided informed consent prior to participation.

## Data Availability

The data supporting the findings of this study are available from the corresponding author upon reasonable request. Due to ethical and privacy considerations, the original raw eye-tracking and interaction log data contain personally identifiable information and cannot be made publicly available. To support transparency and reproducibility, the authors will provide the cleaned and anonymized task-level, interval-level, and bin-level datasets used for the reported analyses, together with data dictionaries and analysis scripts, upon reasonable request.

## Abbreviations

The following abbreviations are used in this manuscript:

AOI	Area of Interest
CSCW	Computer-Supported Cooperative Work
EXP1	Experiment 1 (Drag-and-Drop Interface)
EXP2	Experiment 2 (Dropdown Interface)
EXP2A	Experiment 2, Weekend-Visible Condition
EXP2B	Experiment 2, Weekend-Hidden Condition
ISO	International Organization for Standardization
RQ	Research Question
TOI	Task-Oriented Interval
UI	User Interface

## Appendix A

This appendix provides a design landscape snapshot of mainstream calendar and scheduling products to contextualize the UI factors studied in this paper (interaction grammar and horizon/boundary cues). The purpose is not to review products, but to document that (i) direct manipulation vs. slot-picking interaction styles and (ii) weekend/evening boundary representations are common, consequential design choices in widely used systems (Table A1).

Because product behavior can vary by platform (Web vs. desktop vs. mobile), account tier, and user settings, we use a conservative four-value coding scheme and record platform notes when relevant. We coded each product’s primary scheduling flow and relevant view options on its main interface using a conservative four-value scheme: 1 = supported by default, 0 = not supported by default, C = configurable, and U = unknown/unverified. Because behavior varies by platform, plan, and settings, entries labeled C or U should be read as illustrative rather than definitive.

Product feature snapshot:

1 = supported by default,

0 = not supported by default,

C = configurable,

U = unknown;

Platforms: Web, Win (Windows), macOS, iOS, Andro.

**Table A1:** Mainstream calendar and scheduling-product designs.

Product	Category	Primary Platform (Coded)	Manualentry	AI_Autoschedule	Drag_MoveResize	Dropdown_SlotPick	Weekend_Present	Evening_After18_Present	Weekend_VisualCue	NonWork_VisualCue	WorkingHours_Setting	Booking_AvailabilityMode	Platform Notes
Google Calendar	Grid calendar	Web	1	C	1	0	C	C	U	U	C	C	Behavior depends on Workspace vs. consumer and settings.
Google Calendar—Appointment schedules	Booking/slot-picking	Web	C	0	0	1	C	C	0	0	C	1	Invitees select from available slots; host defines availability.
Microsoft Outlook Calendar	Grid calendar	Web/Win	1	C	C	0	C	1	U	1	1	0	Non-working hours may be visually differentiated; AI depends on Microsoft 365 Copilot Version 30.0.440217001 (Work) availability.
Microsoft Bookings	Booking/slot-picking	Web	C	0	0	1	C	C	0	0	1	1	Weekend/evening depends on business hours/availability settings.
Apple Calendar	Grid calendar	macOS	1	0	C	0	C	C	U	C	C	0	macOS offers workweek/7-day and hour-range display options; mobile differs.
Notion Calendar (Cron)	Grid calendar	macOS/Win	1	0	1	0	C	U	U	U	U	C	Explicit show/hide weekends; database events can be dragged onto calendar.
Fantastical	Grid calendar	macOS/iOS	1	0	1	0	C	C	1	C	C	0	Supports weekend highlighting and day start/end (hour boundaries).
Zoho Calendar	Grid calendar	Web	1	0	1	0	C	U	U	U	C	C	“Work view/workweek” concepts exist; details vary by configuration.
Calendars by Readdle	Grid calendar	iOS	1	0	1	0	U	U	U	U	U	0	Mobile-first; weekend/non-work cues should be checked per version.
Calendly	Booking/slot-picking	Web	C	0	0	1	C	C	0	0	1	1	Host configures available days and time blocks; invitees pick slots.
Doodle	Scheduling poll (slot)	Web	C	0	0	1	C	C	0	0	C	1	Primary interaction is proposing and voting on candidate slots.
Acuity Scheduling (Squarespace)	Booking/slot-picking	Web	C	0	0	1	C	C	0	0	1	1	Invitees pick from available times; host sets availability rules.
Motion	AI scheduling	Web	1	1	U	C	C	C	U	U	C	C	AI layer schedules/reschedules; UI details vary and should be verified.
Reclaim.ai	AI scheduling	Web	1	1	U	C	C	C	U	U	1	C	Explicit working-hour constraints; presentation depends on calendar integration.
Clockwise	AI scheduling/meeting optimization	Web	C	1	U	C	C	C	U	U	1	C	Optimizes meeting times and focus time; UI depends on integrated calendar.
